# Variance-aware penalized panel models for temporal risk detection from wearable sensor data

**DOI:** 10.1186/s12967-026-08652-7

**Published:** 2026-07-17

**Authors:** Zihao Wang, Min Lu

**Affiliations:** Division of Biostatistics, Miller School of Medicine, University of Miami, Miami, FL 33136 USA

**Keywords:** Wearable sensors, Digital biomarkers, Temporal risk detection, Heteroscedasticity, Panel time series, GARCHX, Physiological monitoring

## Abstract

**Background:**

Wearable devices generate continuous, high-resolution physiological data that offer opportunities for real-time assessment of stress, arousal, and early physiological deterioration, but existing pipelines often treat variability as nuisance noise or rely on labeled classifiers. We present a computational approach for subject-adaptive temporal risk detection that explicitly separates conditional mean and variance dynamics in high-dimensional multisubject sensor data.

**Results:**

The proposed penalized panel ARX–GARCHX model integrates subject-specific baselines, shared autoregressive dynamics, sparse multimodal covariate effects, and covariate-dependent volatility. It produces an exceedance-based risk score that estimates the conditional probability of crossing an individualized physiological threshold. Simulation experiments across stable, seasonal, transient-regime, and sustained-regime settings showed that modeling covariate-driven variance improves recovery of threshold-exceedance risk when volatility is structured. In the Wearable Stress and Affect Detection (WESAD) demonstration, the score provided an interpretable, label-free temporal summary that separated stress-associated windows more clearly than raw heart-rate summaries and remained lightweight for streaming use.

**Conclusion:**

Variance-aware penalized panel modeling provides a reproducible methodology for converting noisy wearable streams into subject-adaptive risk-state scores. It is intended for translational feature extraction, with prospective validation required before clinical decision support.

**Trial registration:**

Not applicable. This study is a methodological article that uses publicly available secondary data and does not constitute a clinical trial.

**Supplementary Information:**

The online version contains supplementary material available at https://doi.org/10.1186/s12967-026-08652-7.

## Background

Wearable sensing systems generate continuous multisubject, multimodal physiological streams, including heart rate, electrocardiography (ECG), electrodermal activity (EDA), respiration, body temperature, electromyography (EMG), and acceleration. These signals are increasingly used to study stress, autonomic regulation, sleep-wake patterns, activity, and broader psychophysiological states in settings that are closer to daily life than traditional clinic-based assessment [1–4]. For translational medicine, such data may provide scalable, low-burden measurements that complement clinical assessments and patient-reported outcomes.

A major obstacle is that wearable physiological signals are not simply noisy observations of stable latent states. Their temporal structure is affected by motion artifacts, device placement, context, individual baseline differences, and heteroscedastic variability. Much recent work has focused on supervised machine-learning and deep-learning models for wearable affect recognition and physiological state detection [5–10]. Related signal-processing work has emphasized denoising, adaptive filtering, instantaneous-frequency estimation, forecasting, and anomaly detection for wearable or biomedical sensor streams [11–17]. These approaches demonstrate the value of continuous physiological monitoring, but many rely on dense labels, short-window classification, or feature extraction without explicitly separating changes in location from changes in volatility. Distinguishing changes in location from changes in volatility is important in biomedical and translational studies, where a risk-relevant physiological deviation may depend on a participant’s own baseline and variance pattern rather than on a fixed population threshold [18–20].

Model-based temporal analysis provides a complementary route. Autoregressive models with exogenous covariates (ARX) are useful for dynamic input-output relationships [21], and generalized autoregressive conditional heteroscedasticity models with covariates (GARCHX) allow the conditional variance to depend on lagged variation and external predictors [22–26]. However, standard implementations are usually designed for single series and low-dimensional inputs. Wearable studies, in contrast, often involve repeated measurements from multiple participants and many correlated multimodal features. A translationally useful approach should therefore pool information across participants, retain subject-specific baselines, select informative covariates, and return a risk score that can be interpreted on a probability scale. Dynamic panel modeling [27] and sparse penalization [28–31] provide useful building blocks, but they are rarely integrated with covariate-driven volatility modeling for wearable physiological risk detection. Related work on variance-guided regression for heteroscedastic data has shown that explicitly leveraging conditional variance structure can improve prediction [32]. In many sensing applications, the goal extends beyond forecasting to deriving a *temporal risk score* that captures mean dynamics and variance fluctuations under realistic noise. Wearable streams often show sharp volatility changes driven by motion or context, motivating explicit variance modeling when extracting stable temporal representations from noisy signals.

**Fig. 1:**
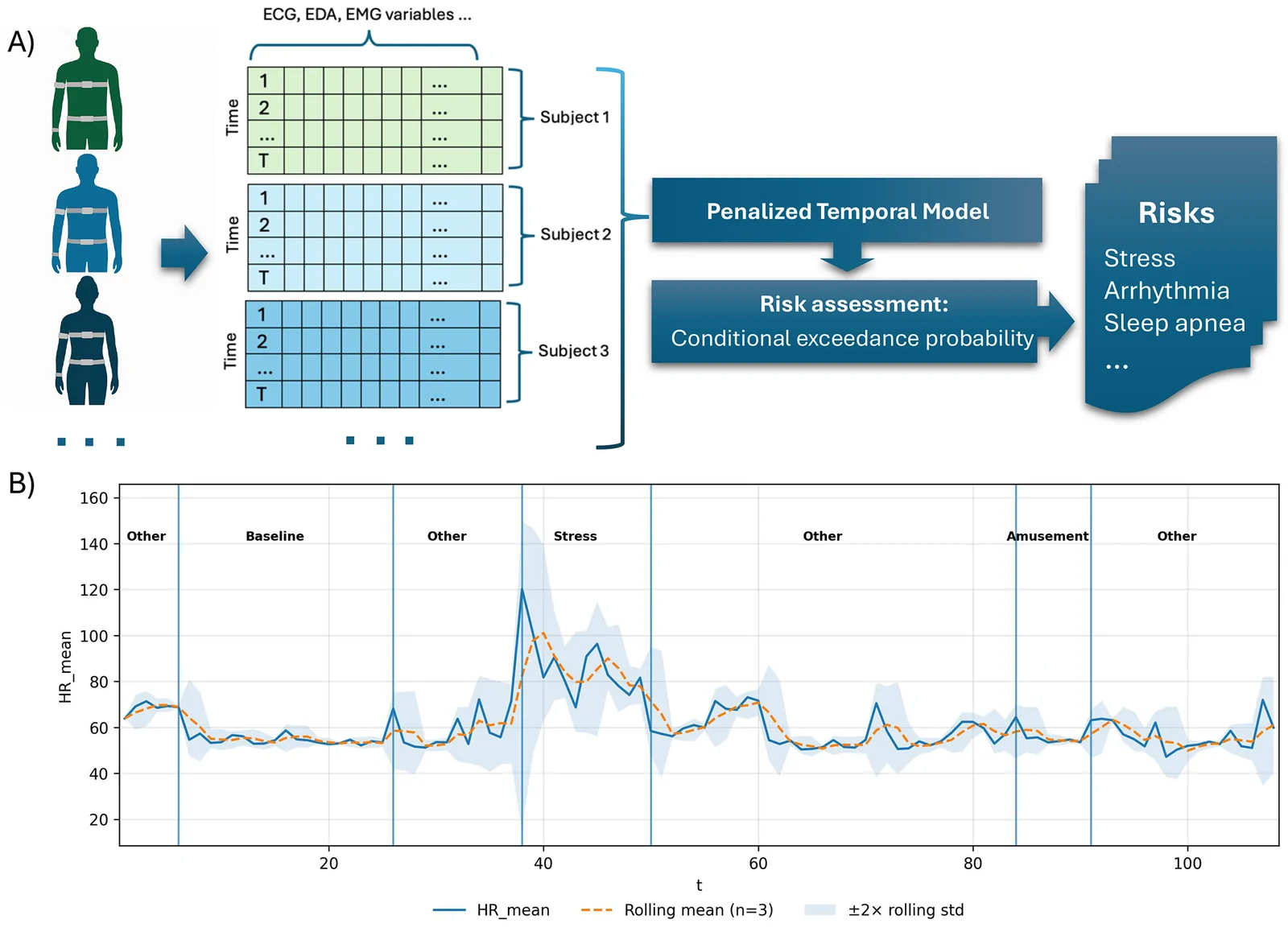
Conceptual workflow for temporal risk detection from wearable sensor data. Multisubject physiological streams are represented as window-level features, analyzed with a penalized temporal model, and summarized through a conditional exceedance probability that can be used as a subject-adaptive risk assessment. Any downstream conditions shown in panel (**A**), such as arrhythmia or sleep apnea, are *hypothetical* illustrative applications and are not analyzed in this study; the only empirical demonstration here is the WESAD stress benchmark. The lower panel illustrates heart-rate dynamics across annotated WESAD segments

We propose a variance-aware penalized panel model for temporal risk detection from wearable sensor data. The model combines a panel ARX mean equation with a GARCHX variance equation, uses sparsity penalties to handle high-dimensional multimodal covariates, and outputs an exceedance-based risk score. The score estimates the conditional probability that a target physiological signal exceeds a subject-specific threshold after accounting for recent history, covariates, and time-varying variance. Figure 1 summarizes the workflow. Panel (A) shows how multimodal wearable streams are processed through the penalized ARX–GARCHX framework to obtain variance-aware temporal representations, while Panel (B) illustrates pronounced volatility changes in windowed heart-rate data from the Wearable Stress and Affect Detection (WESAD) dataset [4]; here HR_mean denotes the mean heart rate (in beats per minute) within each 60-s window, the target signal used throughout.

The aims of this study were: (i) to develop a penalized panel ARX–GARCHX model for high-dimensional multisubject wearable streams; (ii) to define an interpretable exceedance-based risk score; (iii) to evaluate recovery of the risk score in simulations with structured volatility; and (iv) to demonstrate proof-of-concept performance for stress-associated temporal risk detection using the WESAD benchmark dataset [4, 33].

## Methods

### Penalized panel ARX–GARCHX model

Let *s = 1, …, S* index subjects and *t = 1, …, T* index time. All subjects share the same time length *T*, so we consider a balanced panel. We observe $\{(y_{s,t},x_{s,t})\}$, where $y_{s,t}$ is a univariate sensor-derived signal of interest (e.g., heart rate), and $x_{s,t} \in \mathbb{R}^d$ denotes a high-dimensional vector of multimodal covariates (e.g., engineered features or concurrent sensor channels). For the response variable $y_{s,t}$, we consider an autoregressive panel data model [27] of the form

1 $$y_{s,t} = \alpha_s + \sum_{i=1}^{P}\theta_i\,y_{s,t-i} + \sum_{j=0}^{Q} x_{s,t-j}^{\!\top}\beta_j + \sigma_{s,t}\,\varepsilon_{s,t},$$

where $\mathbb{E}[\varepsilon_{s,t}\mid x_{s,t},\mathcal{F}_{t-1}]=0$, $\operatorname{Var}(\varepsilon_{s,t}\mid x_{s,t},\mathcal{F}_{t-1})=1$, and $\mathcal{F}_{t-1}$ denotes the information set generated by all past observations and covariates up to time *t-1*. The innovations $\varepsilon_{s,t}$ thus have zero conditional mean and unit conditional variance given the past, making $\sigma_{s,t}$ the conditional standard deviation. Equivalently, define $\mu_{s,t}:=\alpha_s+\sum_{i=1}^{P}\theta_i\,y_{s,t-i}+\sum_{j=0}^{Q} x_{s,t-j}^{\!\top}\beta_j$ so that $y_{s,t}=\mu_{s,t}+\sigma_{s,t}\varepsilon_{s,t}$.

The conditional variance model is

2 $$ \sigma_{s,t}^2=\omega_s+\sum_{\ell=1}^{L}b_\ell\sigma_{s,t-\ell}^2+\sum_{r=1}^{R}a_r e_{s,t-r}^2+\sum_{m=0}^{M}x_{s,t-m}^{\top}\gamma_m,$$

where $e_{s,t}=y_{s,t}-\mu_{s,t}$ and $\omega_s$ is a subject-specific baseline variance. To ensure $\sigma_{s,t}^2 > 0$, we (i) enforce $a_r,b_\ell\ge 0$, $\omega_s\ge \omega_{\min} > 0$, and (ii) project the linear predictor to a positive set when needed. Implementation safeguards are detailed in Supplementary Section S2.

*Sharing vs. individual effects.* Intercepts $\alpha_s$ (mean) and $\omega_s$ (baseline variance) are *subject-specific*, while $\{\theta_i\},\{\beta_j\},\{\gamma_m\},\{a_r\},\{b_\ell\}$ are *shared* population parameters.

### Penalized quasi-likelihood

Parameters are estimated by penalized Gaussian quasi-likelihood [34]:

3 $$ \min_{\alpha,\theta,\beta,\omega,a,b,\gamma} \sum_{s=1}^{S}\sum_{t=1}^{T}\left\{\log\sigma_{s,t}^2+\frac{(y_{s,t}-\mu_{s,t})^2}{\sigma_{s,t}^2}\right\} +P_{\lambda}(\beta)+P_{\rho}(\gamma),$$

where $P_\lambda$ and $P_\rho$ may be the $\ell_1$, elastic net, smoothly clipped absolute deviation (SCAD) [35], minimax concave penalty (MCP) [36], or group penalties. In this paper, we employ the $\ell_1$ penalty to select informative covariates in both the conditional mean and conditional variance equations. Model fitting uses alternating updates of the mean and variance blocks.

Although the focus of this work is methodological and applied, the proposed penalized estimator enjoys standard high-dimensional guarantees. Under regularity, restricted-strong-convexity, and score-bound conditions on the panel process (Assumptions S1–S3 in the Supplementary Material), the penalized ARX–GARCHX estimator $(\widehat{\beta},\widehat{\gamma})$ attains an excess-risk rate of order $(s_\beta+s_\gamma)\log p/(ST)$, where $(s_\beta,s_\gamma)$ are the sparsity levels of the oracle pair $(\beta^\star,\gamma^\star)$ and *p* is the ambient dimension; the formal statement and full proof are given in Supplementary Section S1 (Theorem S1).

### Algorithm: Alternating penalized estimation

Given current conditional variance estimates, the subject-specific intercepts in the conditional mean equation are updated by weighted least squares, and the shared mean coefficients $(\theta,\beta)$ are obtained from a weighted $\ell_1$-penalized regression. The variance block uses a decoupled update: *(ω,a,b)* is refit by constrained nonlinear optimization with *γ* held fixed, after which *γ* is updated by a proximal-gradient step under positivity safeguards. A forward recursion then refreshes $\sigma_{s,t}^2$ for the next outer iteration. The complete pseudocode (Algorithm S1), positivity enforcement (Remark on the variance recursion), and convergence/tuning details are given in Supplementary Section S2. For all experiments, covariates were standardized within each subject and the lag orders were fixed to *(P,Q,L,R,M)=(1,0,1,1,0)*, reflecting the short-memory structure typical of wearable sensor data; penalties $(\lambda,\rho)$ were selected by blockwise time-series cross-validation that preserves temporal ordering within each subject.

### Exceedance-based risk score

The fitted model provides estimated conditional mean $\widehat{\mu}_{s,t}$ and conditional standard deviation $\widehat{\sigma}_{s,t}$. For a subject-specific threshold $c_s$, we define the temporal risk score as

4 $$ \pi_{s,t}(c_s)=\Pr(Y_{s,t} > c_s\mid\mathcal{F}_{t-1},x_{s,t}) =1-F_{\varepsilon}\left(\frac{c_s-\mu_{s,t}}{\sigma_{s,t}}\right),$$

where $F_{\varepsilon}$ is the innovation distribution function. We used the Gaussian distribution for the primary analysis; a standardized Student-*t* distribution can be used for heavier-tailed signals. Choosing $c_s$ as a participant-specific quantile of the observed signal makes the resulting score comparable across participants while preserving subject-specific baselines.

The score is not a diagnosis. It is an interpretable physiological risk-state indicator: high values indicate that, given the recent history and multimodal covariates, the target signal is likely to exceed an individualized threshold. The score and its interpretation are explicitly conditional on the choice of threshold $c_s$: $\pi_{s,t}(c_s)$ answers “how likely is the signal to exceed $c_s$ right now,” so a different $c_s$ defines a different exceedance event and a different score. The threshold should therefore be chosen to match the intended use—for example a within-subject quantile for baseline-relative monitoring, or a clinically anchored value when one is available—and reported alongside the score. We return to this dependence, and to how $c_s$ is set in practice, in Sections “Model specification and analysis plan” and “Discussion”.

## Simulation studies

### Data generating scenarios

We evaluated the proposed variance-aware autoregressive model on synthetic panel datasets designed to challenge both mean tracking and variance modeling under smooth and piecewise changes. For subjects *s=1,…,S* and times *t=1,…,T*, we generated innovations $\varepsilon_{s,t}$ that were conditionally independent over time given $\mathcal{F}_{t-1}$ and independent across subjects. We considered either Gaussian or standardized Student-*t* innovations,

5 $$\varepsilon_{s,t}\sim\begin{cases}N(0,1), & \mathrm{Gaussian},\\t_\nu/\sqrt{\nu/(\nu-2)}, & \mathrm{Student-}t\ (\nu > 2),\end{cases}\qquad \operatorname{Var}(\varepsilon_{s,t})=1.$$

The exogenous vector $X_{s,t}$ combined a low-dimensional signal component, a binary regime indicator that induced heteroscedasticity, and a high-dimensional block of nuisance noise:

6 $$\begin{aligned}X_{s,t}=&\left(Z_{s,t}^{\top},X_{s,t}^{\mathrm{bin}},W_{s,t}^{\top}\right)^{\top},\quad Z_{s,t}\in\mathbb{R}^{2},\quad \\&X_{s,t}^{\mathrm{bin}}\in\{0,1\},\quad W_{s,t}\in\mathbb{R}^{d_{\mathrm{noise}}}.\end{aligned}$$

The two-dimensional signal block $Z_{s,t}$ carried the true signal. As shown in Table 1, we considered independent bivariate standard normal draws, coordinate-wise autoregressive processes with mild persistence, and a seasonal sine-cosine pair. These generators represent independent baseline variation, short-memory persistence typical of sensor drift, and diurnal or circadian-like variation. The binary regime indicator $X_{s,t}^{\textrm{bin}}$ modulated volatility and was generated either by a within-subject median trigger or by piecewise-constant segments. The median trigger captures personalized volatility bursts, whereas the piecewise design creates sustained regime shifts resembling activity or context changes. The nuisance block $W_{s,t}$ followed a weakly persistent autoregressive process,

7 $$\begin{aligned}W^{(\ell)}_{s,t}=&\rho W^{(\ell)}_{s,t-1}+\xi^{(\ell)}_{s,t},\qquad \\&\xi^{(\ell)}_{s,t}\overset{i.i.d.}{\sim}N(0,1),\qquad \\&W^{(\ell)}_{s,0}\sim N\left(0,\frac{1}{1-\rho^2}\right),\end{aligned}$$

for $\ell=1,\ldots,d_{\textrm{noise}}$. Modeling nuisance covariates as autocorrelated rather than white noise makes separation of true signals from background variation more realistic.

**Table 1:** Simulation design for scenarios S1–S4

	S1	S2	S3	S4
$\omega_s^{\ast}$	0.10	0.10	0.01	0.01
$\theta_i$	0.40	0.50	0.40	0.40
$(a_r, b_\ell)$	(0.05, 0.50)	(0.06, 0.50)	(0.06, 0.20)	(0.06, 0.20)
$\beta_j$ on *Z*	(0.40, 0)	(0.80, 0.60)	(0.20, 0.10)	(0.80, 0.60)
$\gamma_m$ on *Z* or $X^{\mathrm{bin}}$	*Z*: (0.20, 0)	*Z*: (0.10, 0)	$X^{\mathrm{bin}}{:}\,0.40$	$X^{\mathrm{bin}}{:}\,0.50$
Innovations $\varepsilon_{s,t}$	$\mathcal{N}(0,1)$	$\mathcal{N}(0,1)$	$\mathcal{N}(0,1)$	$t_\nu$ ($\nu{=}8$)
Signal *Z*	i.i.d. $\mathcal{N}_2(0,I_2)$	seasonal $(\sin,\cos)^{\S}$	AR(1)	seasonal $(\sin,\cos)^{\S}$
Regime $X^{\mathrm{bin}}$	none	none	$\textrm{subject median}^{\dagger}$	$\textrm{piecewise}^{\ddagger}$
Burn-in	200	220	180	180

All scenarios share $\alpha_s\sim\mathcal{N}(0,0.5^2)$, and the noise generator *W* follows the AR(1) process in Eq. (7)

*Note.* Parameter values are held constant across indices *i*, *r*, *ℓ*, *j*, and *m* within each scenario. The burn-in period is used only during data generation to remove initialization transients and is discarded before model fitting. Consequently, it does not affect the estimation procedure, the estimands, or the reported Bias and RMSE. The modest differences in burn-in lengths across scenarios were chosen for simulation convenience to ensure that the retained observations are approximately from the stationary regime

* $\omega_s$ is set to be the same for all subjects *s*

**Fig. 2:**
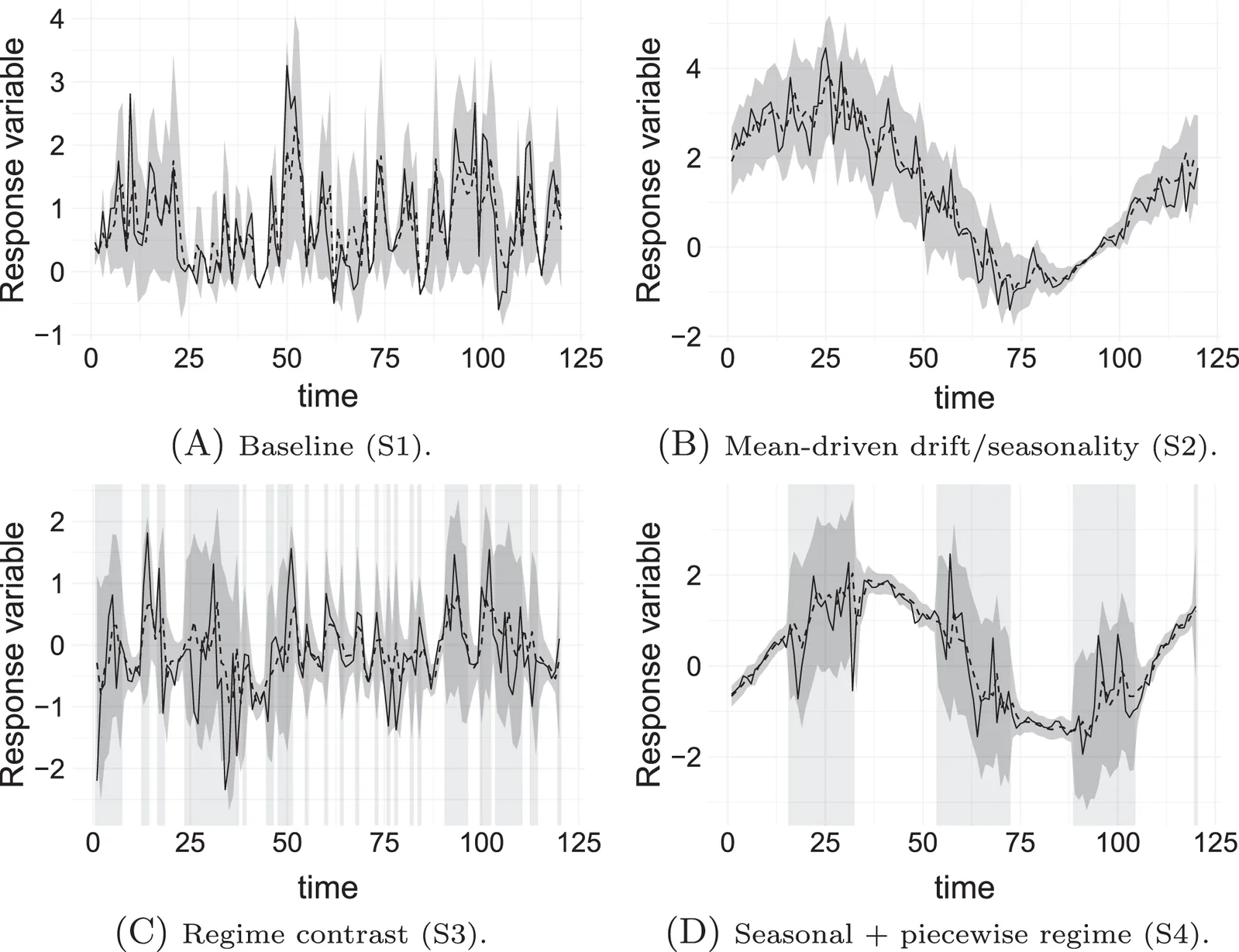
Illustration of one example subject for each scenario (S1–S4) with $y_{s,t}$ (solid), $\mu_{s,t}$ (dashed), and the band $\mu_{s,t}\pm2\sigma_{s,t}$. For scenarios 3 and 4, gray shading denotes intervals where the regime indicator is active ($X^{\textrm{bin}}{=}1$)

*\S* $Z^{(1)}=\sin(2\pi t/T_P)$ and $Z^{(2)}=\cos(2\pi t/T_P)$ are seasonal covariates shown in Fig. 2(B,D)

*†* Subject-median trigger $X^{\mathrm{bin}}_{s,t}=\mathbb{1}\{Z^{(1)}_{s,t} > \textrm{median}_t(Z^{(1)}_{s,t})\}$, computed per subject over the post-burn-in window; this produces frequent, short high-variance bursts

*‡* Piecewise-constant $X^{\mathrm{bin}}_{s,t}$ alternates between 0 and 1, with run lengths jittered around a target scale $T_L$ shared across subjects, yielding sparse, long-duration blocks

As shown in Fig. 2, in Scenario S1 the ribbon varies only modestly and $y_{s,t}$ tracks $\mu_{s,t}$ closely, indicating relatively stable variance without regime-driven shifts. In Scenario S2, sinusoidal covariates generate a smooth diurnal modulation of $\mu_{s,t}$ while ribbon width remains approximately constant, so variability is primarily mean-driven. Scenario S3 has numerous short shaded intervals that coincide with brief increases in $\sigma_{s,t}$: the ribbon widens but $\mu_{s,t}$ remains level, demonstrating transient and variance-driven episodes. In Scenario S4, extended shaded blocks coincide with sustained increases in $\sigma_{s,t}$ and more frequent large deviations, consistent with heavy-tailed innovations.

### Competing models and evaluation metrics

We compare six variants within the ARX–GARCH family (Models A–F). Models A–D include covariates in the conditional mean and differ in whether the mean or variance components are penalized and whether *X* also enters the variance equation; Model E adds the intermediate specification with a penalized mean and an *unpenalized* *X*-dependent variance, and Model F is a covariate-free AR–GARCH baseline:

- **Model A**: ARX–GARCH (variance independent of *X*),
- **Model B**: $\textrm{ARX}_p$–GARCH (penalized mean; variance independent of *X*),
- **Model C**: ARX–GARCHX (variance includes *X*, unpenalized),
- **Model D**: $\textrm{ARX}_p$–$\textrm{GARCHX}_p$ (penalized mean and penalized variance),
- **Model E**: $\textrm{ARX}_p$–GARCHX (penalized mean; unpenalized *X*-dependent variance),
- **Model F**: AR–GARCH (no covariates in the mean or variance; baseline).

Model E isolates the effect of penalizing the variance covariates (E versus D) while holding the penalized mean fixed, and Model F quantifies the value added by covariates over a purely autoregressive, covariate-free specification. Here $\textrm{ARX}_p$ and $\textrm{GARCHX}_p$ denote the ARX mean and GARCHX variance equations with $\ell_1$ penalties applied to the covariate coefficients *β* and *γ*, respectively.

To focus on relevant tails, we evaluate cutoffs *c* at pooled empirical quantiles $p\in\{0.60,0.70,0.80,0.90,0.95\}$, providing global thresholds that preserve between-subject heterogeneity at small *T*. We fix lag orders *(P,Q,L,R,M)=(1,0,1,1,0)* with standardized predictors. From each fitted model, we obtain $(\widehat{\mu}_{s,t},\widehat{\sigma}_{s,t})$ and compute $\widehat{\pi}_{s,t}(c)$. For a given threshold *c*, we compare the estimate $\widehat{\pi}_{s,t}(c)$ with the oracle $\pi_{s,t}(c)$ via

$$\begin{aligned}&\operatorname{Bias}(c)=\frac{1}{ST}\sum_{s=1}^{S}\sum_{t=1}^{T}\bigl(\widehat{\pi}_{s,t}(c)-\pi_{s,t}(c)\bigr),\\&\;\text{and }\operatorname{RMSE}(c)=\sqrt{\frac{1}{ST}\sum_{s=1}^{S}\sum_{t=1}^{T}\bigl(\widehat{\pi}_{s,t}(c)-\pi_{s,t}(c)\bigr)^2}.\end{aligned}$$

We averaged the bias and RMSE across the five cutoff values to summarize each replication. Simulations were conducted with *S=15* subjects, $d_{\textrm{noise}}=100$ noise covariates, and time horizons $T\in\{120,240\}$, chosen to match the scale of our real-data application to the WESAD study. We set *ρ=0.5* for the AR(1) noise variables *W* and *ρ=0.6* for the signal variable *Z* in Scenario 3. In Scenarios 2 and 4, we used a seasonal period $T_P=T/3$ for the covariate *Z*, and Scenario 4 additionally used a piecewise regime run length of $T_L=20$ for the binary covariate $X^{\textrm{bin}}$. Each scenario was replicated 100 times.

### Simulation results

**Fig. 3:**
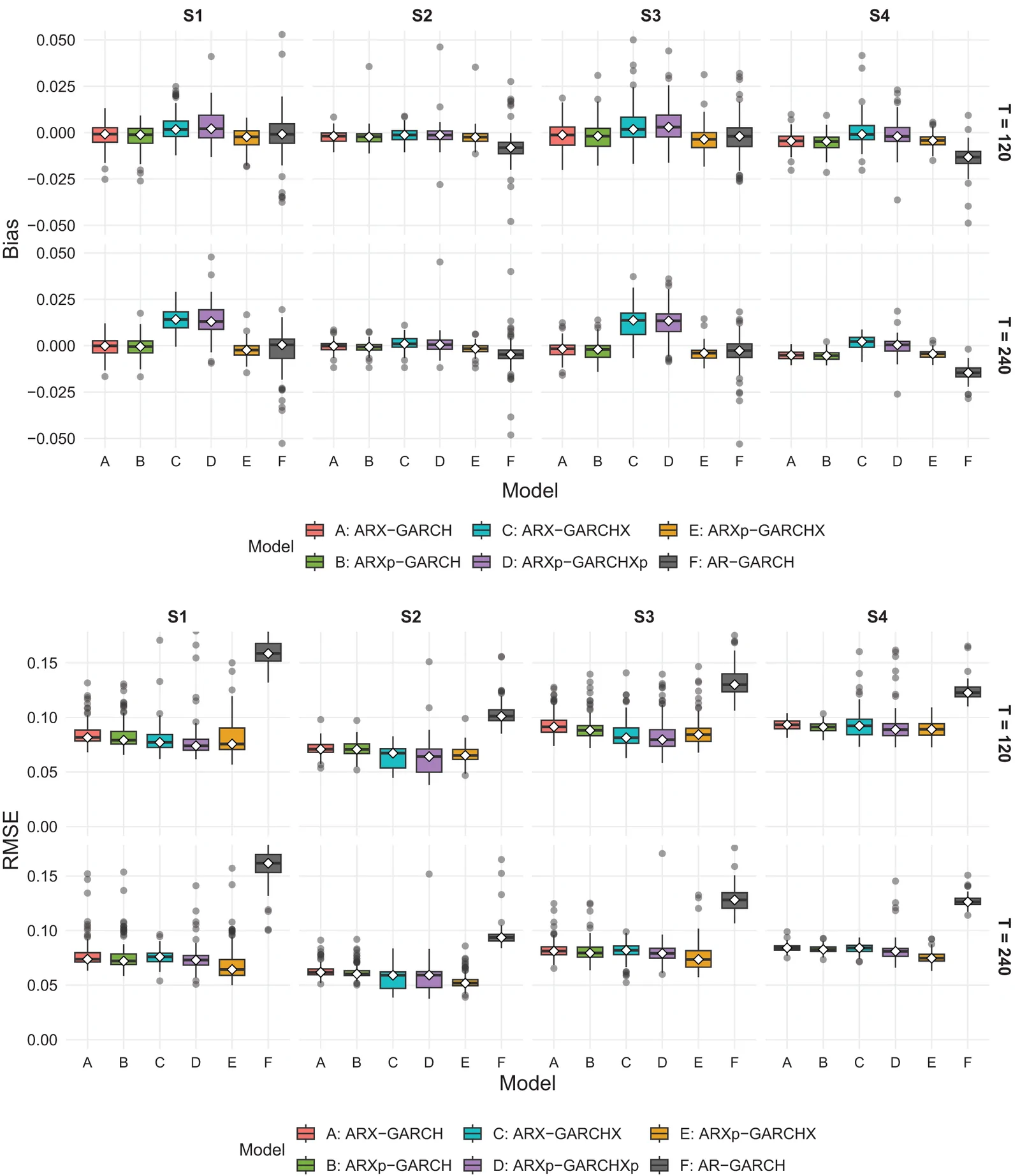
Bias (top) and RMSE (bottom) of the estimated risk score across simulation scenarios S1–S4 and models A–F, at horizons *T=120* and *T=240* (boxplots over 100 replications; the white diamond marks the median; the legend identifies the six model variants). Model A: ARX–GARCH; model B: $\textrm{ARX}_p$–GARCH; model C: ARX–GARCHX; model D: $\textrm{ARX}_p$–$\textrm{GARCHX}_p$; model E: $\textrm{ARX}_p$–GARCHX; model F: AR–GARCH (covariate-free baseline). The covariate-free baseline (F) has by far the highest RMSE in every setting, and the variance-aware models (C, D, E) are the most accurate, with E and D essentially indistinguishable

Across all simulated settings, the accuracy of the exceedance-based risk score is governed by whether the conditional variance is allowed to depend on covariates and whether that dependence matches the structure of the underlying volatility. When volatility is stable (Scenario 1), all six models perform similarly, so modeling covariate-driven variance costs little where it is unnecessary. Once volatility becomes structured—through seasonality (Scenario 2), regime changes (Scenario 3), or the two combined with heavy-tailed innovations (Scenario 4)—the variance-aware specifications separate clearly from the variance-agnostic baselines, attaining lower RMSE and near-zero bias in the risk score (Fig. 3). The proposed specification **D** is strongest in the seasonal, wearable-like regime (Scenario 2), and its margin widens as the regime structure intensifies (Scenarios 3–4) and as the horizon lengthens to $T{=}240$; even under the heavy-tailed innovations of Scenario 4, **C** and **D** remain robust and continue to outperform the variance-agnostic baselines. Extending the comparison to all six models refines rather than overturns this picture. Model **E** ($\textrm{ARX}_p$–GARCHX; penalized mean, unpenalized *X*-dependent variance) is essentially indistinguishable from **D**—indeed the most accurate model at the longer horizon $T{=}240$, with the lowest RMSE in all four scenarios—indicating that penalizing the variance covariates chiefly stabilizes estimation at small *T* rather than shifting the central tendency. At the other extreme, the covariate-free baseline **F** (AR–GARCH) has by far the highest RMSE in every scenario (roughly *0.10*–*0.16* versus *0.05*–*0.09* for the covariate-driven models), quantifying the accuracy lost by ignoring exogenous covariates altogether. Taken together, the six models bracket the value of covariate-driven variance: decisive when volatility is structured, negligible when it is not, and best realized by the penalized specification that couples covariate information with regularized estimation. Full six-model results are reported in Supplementary Table S1.

Both variance-aware models **C** and **D** exhibit a mild positive bias, reflecting slight overestimation of exceedance probabilities, but the mechanism differs between them. In Model **D**, which applies an $\ell_1$ penalty to the variance-covariate coefficients *γ*, the bias reflects shrinkage of *γ* toward zero, which mildly attenuates the fitted conditional variance and shifts exceedance probabilities upward through the curvature of $z\mapsto 1-\Phi(z)$. Model **C**, by contrast, applies *no* penalty in the variance equation; its bias instead arises from finite-sample estimation noise when the high-dimensional variance-covariate vector is fit without regularization (many near-zero coefficients estimated from short series), together with the positive-variance safeguard, which together inflate small fitted variances. In both cases the effect is small. Even so, their RMSE remains lowest overall, indicating that the variance-aware formulations achieve a favorable bias–variance tradeoff and provide the most reliable exceedance-based temporal summaries across scenarios and time horizons. Replication-level Bias and RMSE means with standard errors are tabulated in Supplementary Table S1.

## The WESAD lab study

We evaluate the proposed framework using the WESAD dataset [4, 33], a controlled multimodal wearable-sensing benchmark involving 15 adult participants. Each subject completed a scripted session consisting of several annotated segments (e.g., resting, task-engaged, and amusement periods), providing clear temporal structure for analysis. Participants wore two devices—a chest-worn RespiBAN and a wrist-worn Empatica E4—so all physiological streams are time-synchronized across modalities.

The chest device provides ECG, EDA, EMG, respiration, skin temperature, and 3-axis acceleration, while the wrist device provides photoplethysmography (PPG/BVP), EDA, temperature, and acceleration. Because the protocol follows a predefined sequence of segments and the recordings are concurrent across modalities, WESAD offers clean annotations and consistent multisensor coverage suitable for multisubject or subject-wise analysis.

In our study, we focus on the chest-worn streams, as they provide the most precise heart-rate estimation. Heart rate serves as the target signal for temporal modeling due to its continuity, stability under windowing, and sensitivity to motion and other factors that drive heteroscedastic behavior in wearable data. The remaining modalities (EDA, EMG, RESP, ACC, TEMP) are incorporated as covariates to inform the covariate-driven mean and variance dynamics within the proposed variance-aware temporal risk model. The dataset annotations are used only for visualization and reference-based evaluation, not for model fitting.

### Data preparation

Preprocessing and feature extraction were performed in Python using toolboxes designed for physiological-signal analysis. NeuroKit2 was used for ECG/PPG peak detection and for deriving HR, heart-rate variability (HRV), respiration, and EDA features [37]. For chest-worn devices, all streams were segmented into fixed 60-s windows with a 60-s hop. A window was retained only if at least 80% of its samples were finite across the required channels. To ensure temporal alignment across modalities, the raw streams were anti-aliased and resampled onto a common low-rate timeline prior to computing windowed features and cross-modal correlations.

Feature construction was carried out per 60-s window. From the synchronized multimodal series, we computed (i) per-series moments (mean, standard deviation, skewness, and excess kurtosis) and (ii) all pairwise Pearson cross-modal correlations to capture coordinated multimodal changes, a common pattern in wearable sensing where channels co-modulate under shared motion or environmental drivers. This produced a larger set of candidate window-level features, which were reduced to *d=77* by removing one member of each pair of highly collinear features (absolute Pearson correlation above *0.90*, using caret::findCorrelation); the retained *77* features enter the model and are the covariates listed in Table 2. Windows with fewer than *80%* finite samples in a required channel were dropped before feature construction, and any window with a non-finite feature value after construction was excluded; no imputation was used. After these steps, $1{,}427$ windows remained across the *15* participants (*87*–*117* windows per participant; participants S1 and S12 are excluded following the WESAD convention). The complete list of the *77* features is given in Supplementary Section S8 (Supplementary Table S6), and the retained window count for each participant is given in Supplementary Table S3. Because participants contribute different numbers of windows, the analyzed panel is in fact *unbalanced*; the balanced-panel notation of Section “Penalized panel ARX–GARCHX model” is used only for expositional clarity, and the estimator constructs all lags within each participant separately (see Supplementary Section S2), so unequal series lengths are handled without truncation or padding.

**Table 2:** Chest-worn multimodal features used in the models (60 s windows)

Block	Summary of features
ECG / HRV	Short-term variability (RMSSD/pNNx), LF/HF power and ratio, simple Poincare geometry, and compact entropy/complexity indices
Respiration & BRV	Breathing rate and amplitude (levels and variability), inspiratory/expiratory durations, duty cycle, and breath-to-breath variability
EDA	Tonic level (mean/dispersion/trend) and phasic activity (SCR count, typical amplitude, cumulative phasic area)
EMG	Broadband power, median frequency, spectral entropy, and zero-crossing rate
Accelerometry	Movement intensity (RMS) and dynamics (jerk), axis medians, high-activity tail (P90), plus overall SD/skewness/kurtosis
Skin temperature	Level, per-minute slope (trend), and detrended variability
Cross-channel correlations	Short-lag correlations among RESP, EDA, ACC, TEMP, and EMG to capture coordinated multimodal changes

Summaries are grouped by sensing block

*Note.* RMSSD: root mean square of successive differences; pNNx: percentage of successive NN intervals differing by more than *x* ms; LF/HF: low-/high-frequency power ratio; BRV: breathing rate variability; SCR: skin conductance response; RMS: root mean square; SD: standard deviation; RESP: respiration; ACC: accelerometer; TEMP: skin temperature

For reference-based evaluation, each window was also assigned a dataset annotation (baseline, stress, amusement, or transition) using majority vote over the WESAD labels. These annotations were not used when estimating the variance-aware panel model but served only for visualization and for assessing whether the derived exceedance-based risk score provides clearer temporal differentiation than the raw heart-rate values.

### Model specification and analysis plan

We model the 60-s windowed heart-rate mean (HR_mean), denoted $y_{s,t}$, as a stable, continuous signal that captures temporal fluctuations in wearable data. An ARX–GARCHX model with subject-specific intercepts and contemporaneous covariates in both the mean and variance components is fitted according to Eqs. (1) and (2). The lag orders are fixed at *(P,Q,L,R,M)=(1,0,1,1,0)*, a structure suitable for short-memory wearable streams, and $\ell_1$ regularization is applied to $(\beta,\gamma)$ to promote parsimony and mitigate collinearity among the multimodal features.

To provide a normalized exceedance-based risk score across subjects, we evaluate the conditional exceedance probability (4) at subject-specific thresholds. For a chosen quantile *p*, we set $c_s = c_s(p) := \operatorname{Quantile}_p(\{y_{s,t}\}_t)$ – the *p*-th quantile of subject *s*’s observed signal – and substitute this $c_s$ into (4) under the assumed innovation distribution (Gaussian or standardized *t*). For the WESAD analysis we set *p=0.70*, so that $c_s(0.70)$ is the 70th percentile of each participant’s observed HR_mean series; this individualized threshold makes the score comparable across participants while preserving subject-specific baselines. The resulting exceedance probabilities summarize the joint mean–variance dynamics in a compact, interpretable form that is independent of downstream labels.

To clarify the interpretation and estimation of the proposed score, $\widehat{\pi}_{s,t}(c_s)$ denotes the estimated conditional probability that HR_mean exceeds the individualized threshold $c_s$; it is *not* a probability of stress or of any clinical event. Because the conditional variance model uses contemporaneous covariates $x_{s,t}$ (lag order $M{=}0$), $\widehat{\pi}_{s,t}$ represents the current window rather than a one-step-ahead forecast. A forecasting variant can be obtained by restricting both the mean and variance equations to strictly lagged covariates. In this demonstration, the threshold $c_s$, the within-subject covariate standardization, and the subject-specific parameters $(\alpha_s,\omega_s)$ are all derived from the analyzed recordings. In a prospective deployment, these quantities would instead be obtained during an initial subject-specific calibration period and then held fixed during subsequent monitoring. Specifically, $c_s(0.70)$ is defined as the 70th percentile of each subject’s HR_mean distribution over the full recording. It is therefore computed without using any stress annotations, although it is estimated from the entire recording, including the windows that are later used for evaluation. A sensitivity analysis that instead estimates $c_s$ from a leak-free calibration period is reported in Supplementary Section S6 and summarized in Section “Results and subject-level insights”.

#### Evaluation endpoint

For reference-based evaluation, we define a binary window-level endpoint. The positive class consists of windows annotated as *stress* (WESAD label 2), whereas the negative class pools all other retained windows (baseline, amusement, and transition/other). Transition and amusement windows are therefore treated as non-stress rather than discarded. After preprocessing, $1{,}427$ windows from *15* participants were available (*182* stress and $1{,}245$ non-stress); the corresponding per-participant window and class counts are reported in Supplementary Table S3. The annotations are used only for evaluation and never for model fitting.

For comparison with supervised baselines, we implemented logistic regression, random forest, and gradient boosting using standard R packages (stats::glm [38], randomForest::randomForest [39], and gbm::gbm [40]). The supervised classifiers were trained and evaluated under leave-one-subject-out (LOSO) cross-validation, whereas the proposed exceedance score is unsupervised and was evaluated in-sample (fitted once to each participant’s full recording and scored on the same windows). Because the proposed score and the supervised classifiers are not evaluated under a common held-out design, the supervised AUCs are presented as *contextual benchmarks* that illustrate the performance of purely discriminative window-level methods, rather than as a formal head-to-head comparison. The intended primary comparison for the proposed score is against the raw HR_mean baseline, which is evaluated under the same experimental design. These ensemble methods have shown strong discriminative performance in related translational classification tasks [41], but they do not model temporal dependence or subject-level baselines, in contrast to the proposed ARX–GARCHX risk-scoring model.

### Results and subject-level insights

**Fig. 4:**
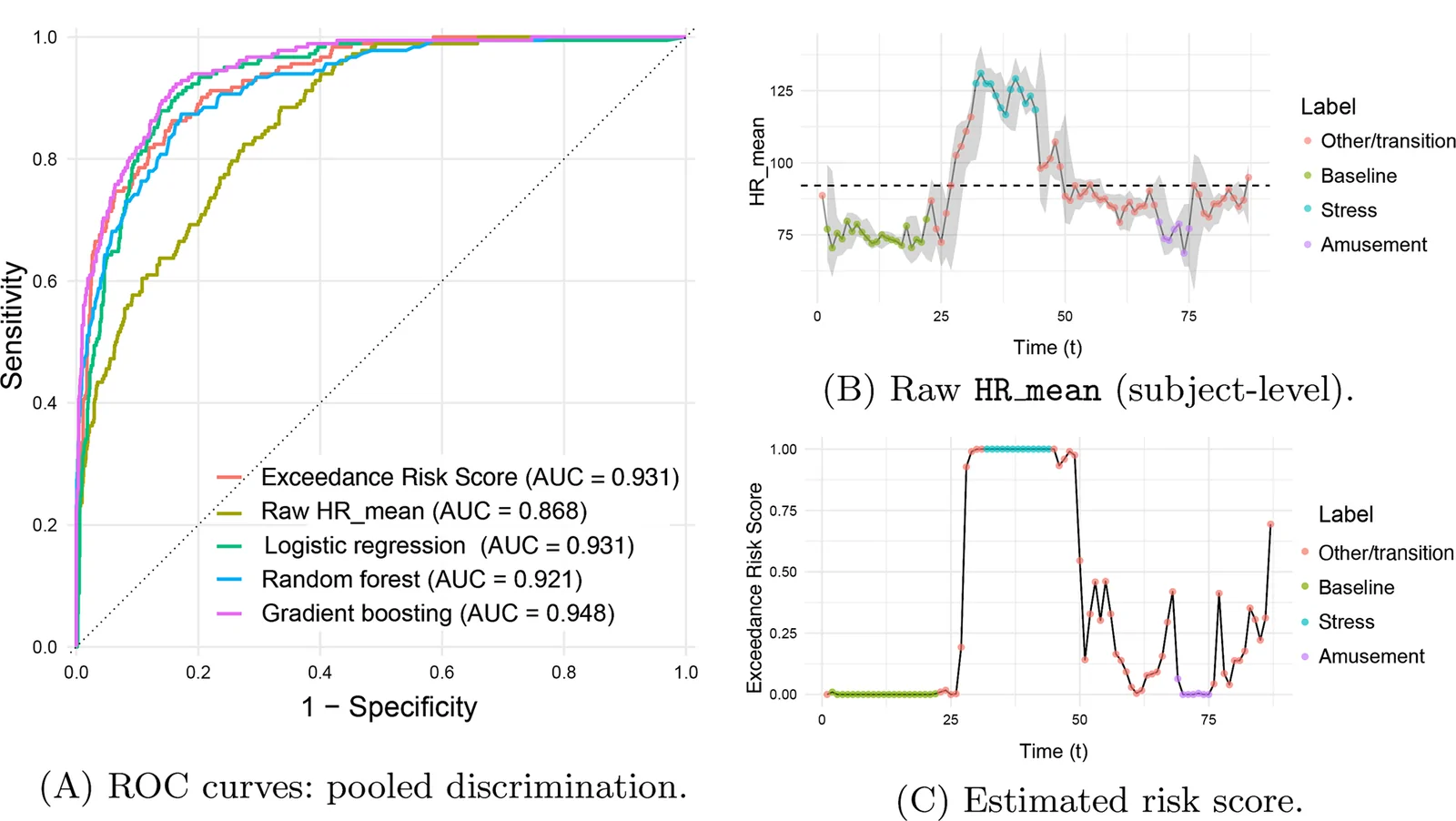
WESAD results. (**A**) ROC curves comparing the variance-aware exceedance-based risk score with the raw 60-s heart-rate mean (HR_mean) and several supervised window-level baselines. The risk score and HR_mean are *unsupervised* (no annotation information used during model fitting) and evaluated in-sample, whereas logistic regression, random forest, and gradient boosting are *supervised* classifiers trained and evaluated using leave-one-subject-out cross-validation and shown as contextual benchmarks; the unsupervised risk score achieves AUC *0.931* (participant-clustered bootstrap *95%* CI *[0.887,0.972]*) and substantially improves upon the raw HR_mean baseline (*0.868*; difference *95%* CI *[0.031,0.094]*). (**B**) Subject-level raw 60-s hr_mean for one representative participant with the subject-specific 70% threshold $c_s(0.70)\approx 92.10$ shown as a dashed line. (**C**) The corresponding estimated risk score over time, showing elevated values during the annotated stress-associated segment and near-zero values elsewhere

Using subject-specific thresholds $c_{s}(0.70)$ and Gaussian innovations, we evaluated the exceedance-based risk score $\widehat{\pi}_{s,t}$ against the raw 60-s heart-rate means (HR_mean). The pooled Receiver Operating Characteristic (ROC) analysis in Fig. 4(A) shows that the variance-aware risk score provides substantially stronger separation across the annotated WESAD segments. The estimated AUC for the risk score was $\textrm{AUC}_{\mathrm{ex}}=0.931$, compared with $\textrm{AUC}_{\mathrm{HR}}=0.868$ for the raw heart-rate signal (*Δ=0.063*). Because repeated windows are clustered within only *15* participants, the pooled DeLong test may overstate precision. We therefore additionally report participant-clustered bootstrap confidence intervals obtained by resampling whole participants ($3{,}000$ bootstrap replicates). This yields *95%* confidence intervals of *[0.887, 0.972]* for $\textrm{AUC}_{\textrm{ex}}$ and *[0.821, 0.918]* for $\textrm{AUC}_{\textrm{HR}}$, while the paired AUC difference has a *95%* confidence interval of *[0.031, 0.094]*, excluding zero. The improvement of the variance-aware risk score over the raw heart-rate signal is thus robust to within-participant clustering, indicating that modeling both mean and variance dynamics yields a more discriminative temporal representation than relying on raw heart-rate measurements alone.

For reference-based benchmarking, we also compared the proposed *unsupervised* metric with several *supervised* classifiers trained on window-level features using leave-one-subject-out cross-validation. Under LOSO cross-validation, logistic regression achieved $\textrm{AUC}_{\mathrm{logit}}=0.931$, random forest achieved $\textrm{AUC}_{\mathrm{RF}}=0.921$, and gradient boosting achieved $\textrm{AUC}_{\mathrm{GBM}}=0.948$. These supervised models use the annotations during training and are evaluated on held-out subjects, whereas the proposed score is unsupervised and evaluated in-sample; the two are therefore not evaluated under a common design, and we present the supervised values as contextual benchmarks rather than as a formal head-to-head comparison. Read in that light, the variance-aware score—estimated without any annotations—attains discrimination in the same range as these purely discriminative window-level classifiers, supporting its ability to extract informative temporal structure from multimodal wearable streams.

Two additional analyses examine the robustness of our results and identify the factors contributing to the observed performance. First, replacing the full-recording threshold $c_s(0.70)$ with a leak-free threshold estimated solely from an early burn-in window reduces $\textrm{AUC}_{\mathrm{ex}}$ from *0.931* to approximately *0.880*; notably, this remains at or above the raw HR_mean baseline (details in Supplementary Section S6). Second, we conduct a focused ablation to isolate the impact of covariate-driven variance by comparing Model B (no covariates in the variance equation) to Model D (the full model); this analysis, along with a calibration assessment for the exceedance event $\{y_{s,t} > c_s(0.70)\}$, is reported in Supplementary Section S7. Both variance-aware models substantially outperform the raw HR_mean baseline and perform comparably to each other. This indicates that the real-data advantage is primarily driven by variance-aware modeling and is robust to the inclusion of covariates in the variance equation, whereas the benefits of covariate-driven variance are more pronounced in the structured-volatility simulation regimes.

It is worth noting that the supervised baselines treat all windows as independent samples and do not incorporate subject-specific baselines or temporal dependence. They therefore serve as strong discriminative benchmarks but do not capture the autoregressive or heteroscedastic structure exploited by the proposed temporal risk-scoring framework.

To complement the aggregate ROC analysis and highlight subject-level dynamics, we examine a representative individual trajectory. Figure 4(B) shows the raw 60-s HR_mean series for a representative participant (WESAD subject S11), with the subject-specific 70% threshold $c_s(0.70)\approx 92.10$ indicated by a dashed line. This participant was selected before qualitative inspection of the model output as a representative case whose protocol contains a clearly delineated stress segment; trajectories for all participants are provided in the replication materials. The annotated WESAD segments reveal a sustained elevation in heart rate during one region of the protocol, with lower variability elsewhere. Occasional excursions approach the threshold outside this segment but do not persist.

Figure 4(C) displays the corresponding risk score $\widehat{\pi}_{s,t}$. The score increases sharply at the onset of the high-variability segment, remains elevated throughout its duration, and drops promptly at its conclusion. Outside this interval, the score remains near zero despite moderate fluctuations in the raw signal. This behavior indicates that the variance-aware temporal model concentrates exceedance probability in periods characterized by sustained deviations relative to the subject’s own baseline while suppressing short-lived fluctuations. Together, the two right-hand panels illustrate that the exceedance formulation provides a subject-adaptive, interpretable temporal summary that aligns closely with the annotated structure of the signal.

The risk score offers a principled and interpretable way to summarize temporal deviations in wearable sensor streams. Its probabilistic range between 0 and 1 provides a normalized scale that is comparable across subjects and robust to individual baseline differences. Unlike raw heart-rate thresholds, the score is sensitive to sustained departures yet resistant to transient fluctuations, yielding a stable temporal characterization of signal patterns. Importantly, the score is estimated without using any annotations, yet it naturally differentiates periods of elevated and quiescent activity in the WESAD protocol. This highlights its utility as an unsupervised temporal feature, suitable both for standalone interpretation and as a noise-robust, low-dimensional input to downstream sensing or machine-learning models.

### Computational feasibility

The WESAD analysis involved fifteen participants, approximately one hundred twenty windows per participant, and seventy-seven covariates. With twenty outer iterations, the mean-update block ran in roughly *0.01* s per iteration and the variance-update block in roughly *0.12* s per iteration, so end-to-end training completed in approximately *26.6* s. After training, a new-window score was computed with sub-millisecond latency. Detailed timings, asymptotic complexity (*O(STd)* for the mean recursion and *O(ST(L+R+M))* for the variance recursion), memory footprint, and hardware information are reported in Supplementary Table S2 (Supplementary Section S4). These results support potential use as a lightweight feature-extraction or monitoring component, although clinical deployment would require prospective validation.

## Discussion

This study reframes variance-aware wearable-sensor modeling as a translational temporal risk-detection problem. The main finding is that explicitly modeling conditional variance, rather than treating variability as nuisance noise, yields an interpretable subject-adaptive score that improves discrimination of stress-associated physiological windows relative to raw heart-rate summaries. In WESAD, the risk score achieved an AUC of 0.931 without using labels during model fitting—compared with 0.868 for the raw heart-rate mean, a difference that remains significant under a participant-clustered bootstrap—while attaining discrimination in the same range as supervised logistic-regression, random-forest, and gradient-boosting classifiers evaluated as contextual benchmarks, and remaining computationally lightweight. We caution that the supervised classifiers were trained and evaluated under leave-one-subject-out cross-validation whereas the unsupervised score was evaluated in-sample, so this correspondence is contextual rather than a formal head-to-head comparison.

The method has several translationally relevant properties. First, it produces a probability-scale score between 0 and 1, which is easier to interpret than arbitrary transformed features. Second, it uses individualized thresholds, making the score more robust to baseline differences across participants. Third, it incorporates multimodal covariates into both the mean and variance equations, allowing the model to distinguish sustained physiological shifts from transient fluctuations. Fourth, it can be computed rapidly after training, which is important for real-time or near-real-time wearable monitoring.

The approach also complements supervised machine learning. Deep learning and sequence models may achieve strong classification accuracy when large labeled datasets are available [42, 43], but many translational applications lack dense, reliable labels. The proposed score can be used as an unsupervised or weakly supervised feature that summarizes subject-specific risk dynamics before downstream prediction. It may therefore be useful as a digital biomarker component in studies of stress, autonomic dysregulation, symptom exacerbation, or other temporally dynamic states. The same idea may also connect with broader time-series segmentation and change-detection methods for multivariate data [44].

Several limitations should be emphasized. The WESAD analysis is a controlled laboratory benchmark with a small number of participants and should not be interpreted as clinical validation. Stress labels in WESAD represent protocol-defined affective states rather than clinically diagnosed stress disorders. External validation in larger and more diverse cohorts is needed before any medical claim can be made. In addition, the supervised classifiers were evaluated under leave-one-subject-out cross-validation, while the risk score was fitted without labels and evaluated post hoc; a fully prospective validation should freeze preprocessing, tuning, thresholds, and model parameters within an outer held-out-subject or external-cohort protocol. The current model also uses fixed lag orders and a balanced-panel formulation. Irregular sampling, missingness, longer-range temporal dependence, and nonlinear mean-variance effects remain important extensions.

Finally, the risk score is threshold-dependent. While we used the 70th percentile of each participant’s heart-rate distribution for interpretability and cross-participant comparison, alternative thresholds may be better suited for different clinical or behavioral endpoints. Because this quantile is computed over the entire recording, it inherently incorporates the same elevated heart-rate windows that later define the positive class. Although no labels are used, this may introduce a modest optimistic bias to the reported discrimination. A sensitivity analysis fixing $c_s$ from a leak-free early burn-in window (Supplementary Section S6) reduces $\textrm{AUC}_{\mathrm{ex}}$ from *0.931* to approximately *0.880* but remains at or above the raw heart-rate baseline. This confirms that the temporal signal is genuine while emphasizing that the exact magnitude of performance is sensitive to threshold selection. In future translational applications, thresholds should be fixed prospectively from an initial calibration period and chosen from clinical anchors, patient-reported outcomes, or adverse-event definitions, rather than relying solely on empirical quantiles computed over the full recording.

## Conclusions

A penalized panel ARX–GARCHX model can convert high-dimensional wearable physiological streams into an interpretable, subject-adaptive temporal risk score. In simulations, variance-aware modeling improved recovery of threshold-exceedance risk under structured volatility. In the WESAD benchmark, the score improved stress-associated window discrimination relative to raw heart rate and performed competitively with supervised baselines while requiring less than 1 ms for new-window inference after training. The method is a promising digital biomarker workflow for translational wearable studies, but prospective external validation is required before clinical use.

## Electronic supplementary material

Below is the link to the electronic supplementary material.

Supplementary Material 1: The Supplementary Material is organized into eight sections. Supplementary Section S1 (*Theoretical Results*) states the regularity, restricted-strong-convexity, and score-bound assumptions and proves a finite-sample oracle inequality (Theorem S1) showing that the penalized estimator achieves an excess-risk rate of order $(s_\beta+s_\gamma)\log p/(ST)$. Supplementary Section S2 (*Algorithmic Details*) gives the full estimation pseudocode (Algorithm S1) for the penalized panel ARX–GARCHX model, including the weighted closed-form mean update, the decoupled *(ω,a,b)* and *γ* variance updates, positivity safeguards, and tuning conventions. Supplementary Section S3 (*Supplementary Simulation Results*) reports the replication-level Bias and RMSE summaries for Models A–F under Scenarios S1–S4 at $T\in\{120,240\}$ in Supplementary Table S1. Supplementary Section S4 (*Supplementary Computational Results*) reports the runtime profile, asymptotic complexity, memory footprint, and hardware specification for the WESAD analysis in Supplementary Table S2. Supplementary Section S5 (*WESAD Endpoint and Per-Participant Composition*) defines the evaluation endpoint, tabulates per-participant window and class counts (Table S3), and reports participant-clustered bootstrap confidence intervals. Supplementary Section S6 (*Sensitivity to the Threshold Choice*) reports the leak-free threshold analysis. Supplementary Section S7 (*WESAD Variance-Covariate Ablation*) compares Model B and Model D on discrimination and calibration of the exceedance event. Supplementary Section S8 (*Reproducibility Details*) lists the complete *77*-feature set (Table S4), retained windows, quality control, tuning grid, temporal folds, and software/code provenance

## Data Availability

The WESAD dataset analyzed in this study is publicly available through the UC Irvine Machine Learning Repository and is described in the original dataset publication [4, 33].

## Abbreviations

ACC: Acceleration
ARX: Autoregressive model with exogenous covariates
AUC: Area under the receiver operating characteristic curve
BRV: Breathing-rate variability
ECG: Electrocardiogram
EDA: Electrodermal activity
EMG: Electromyography
GARCHX: Generalized autoregressive conditional heteroscedasticity model with exogenous covariates
HR: Heart rate
HRV: Heart-rate variability
QMLE: Quasi-maximum-likelihood estimation
RESP: Respiration
RMSE: Root mean squared error
ROC: Receiver operating characteristic
WESAD: Wearable Stress and Affect Detection

## Code availability

An R package implementing the proposed framework, varGuidTS, is available at https://cloud.r-project.org/web/packages/varGuidTS/. Additional source code and replication materials for the analyses reported in this paper are available at https://github.com/zionwzz/variance-guided-risk-demo.

## Declarations

### Reporting guidelines

Not applicable in the formal sense. This is a methodological article using publicly available secondary data; established clinical reporting checklists such as TRIPOD (for prediction-model development and validation) and STARD (for diagnostic accuracy) do not directly apply because the analysis was a proof-of-concept feature-extraction demonstration on a public benchmark, with no prospective enrollment, diagnostic claim, or external clinical validation. Where relevant, we have followed the spirit of the TRIPOD-AI items by reporting the data source, preprocessing pipeline, model specification, tuning procedure, evaluation metrics, intended use, and limitations.

### Use of AI-assisted tools

During preparation of this journal-specific draft, the authors used an AI language model to assist with editorial restructuring and wording. The authors reviewed and are responsible for all scientific content, analyses, interpretations, and conclusions.
