# Supplementary material for: Variance-aware penalized panel models for temporal risk detection from wearable sensor data
Source: J Transl Med. 2026 Jul 17;24:1292. doi: 10.1186/s12967-026-08652-7 (PMC13660148; doi:10.1186/s12967-026-08652-7)
Supplement: Supplementary file 1 [file 12967_2026_8652_MOESM1_ESM.pdf]

SUPPLEMENTARY MATERIAL

Variance-Aware Penalized Panel Models for Temporal Risk Detection from Wearable Sensor Data

S1 Theoretical Results

Under standard smoothness, dependence, and identifiability conditions (Assumptions S1–S3 below), the penalized ARX–GARCHX estimator $(\widehat{\beta}, \widehat{\gamma})$ attains estimation error, up to a constant factor, comparable to that of the best sparse oracle choice of (β, γ). In particular, the estimator correctly identifies the leading covariates in both the mean and variance equations and yields stable mean–variance estimates in high-dimensional multisubject settings. The rest of this section formalizes the assumptions, states the oracle inequality (Theorem S1), and gives a self-contained proof.

Assumption S1 (Regularity and dependence)**.** *The panel process $\{(y_{s,t}, x_{s,t})\}$ satisfies standard mixing and moment conditions, and the loss $\ell_{s,t}(\beta, \gamma)$ is Lipschitz and twice differentiable in (β, γ) with bounded derivatives. These conditions ensure that $R(\beta, \gamma) = \mathbb{E}[\ell_{s,t}(\beta, \gamma)]$ is well defined and smooth, and that empirical-score deviations concentrate at rate $\sqrt{(\log p)/(ST)}$, where p denotes the ambient dimension of the penalized parameters. In our setting, $x_{s,t} \in \mathbb{R}^d$, and the penalized mean and variance coefficients (β, γ) each lie in $\mathbb{R}^d$, so p is of order d.*

Assumption S2 (Restricted strong convexity (RSC))**.** *There exist constants $\kappa > 0$ and $\tau \geq 0$ such that, for all $(\Delta_\beta, \Delta_\gamma)$ in the usual cone associated with the oracle supports $S_\beta = \operatorname{supp}(\beta^\star)$ and $S_\gamma = \operatorname{supp}(\gamma^\star)$,*

$$\big\langle \nabla R(\beta^\star + \Delta_\beta, \gamma^\star + \Delta_\gamma) - \nabla R(\beta^\star, \gamma^\star), (\Delta_\beta, \Delta_\gamma) \big\rangle \geq \kappa(\|\Delta_\beta\|_2^2 + \|\Delta_\gamma\|_2^2) - \tau \frac{\log p}{ST}.$$

Assumption S3 (Score bound)**.** *The empirical score at the oracle pair satisfies*

$$\left\| \frac{1}{ST} \sum_{s,t} \nabla_{\beta,\gamma} \ell_{s,t}(\beta^\star, \gamma^\star) \right\|_\infty \le c_0 \sqrt{\frac{\log p}{ST}}$$

with probability at least $1 - Ce^{-c \log p}$ *for constants* $c, C > 0$.

Theorem S1 (Oracle inequality for penalized ARX–GARCHX)**.** *Let* $(\widehat{\beta}, \widehat{\gamma})$ *minimize the penalized objective*

$$(\widehat{\beta}, \widehat{\gamma}) \in \arg\min_{\beta,\gamma} \Big\{ \mathcal{Q}_{S,T}(\beta, \gamma) := \frac{1}{ST} \sum_{s=1}^{S} \sum_{t=1}^{T} \ell_{s,t}(\beta, \gamma) + \lambda_\beta \|\beta\|_1 + \lambda_\gamma \|\gamma\|_1 \Big\}, \tag{1}$$

and let $(\beta^\star, \gamma^\star)$ *be the oracle sparse pair with* $\|\beta^\star\|_0 \le s_\beta$ *and* $\|\gamma^\star\|_0 \le s_\gamma$. *Under Assumptions S1–S3, and for tuning parameters* $\lambda_\beta, \lambda_\gamma \asymp \sqrt{(\log p)/(ST)}$, *we have, with probability at least* $1 - C \exp(-c \log p)$,

$$R(\widehat{\beta}, \widehat{\gamma}) - R(\beta^\star, \gamma^\star) \;\lesssim\; (s_\beta + s_\gamma) \frac{\log p}{ST}, \tag{2}$$

where the hidden constant depends only on (κ, τ, c_0).

Proof. The proof follows a standard high-dimensional M-estimation argument, adapted to the panel time-series setting via the assumptions on dependence and RSC.

Step 1: Basic inequality. By definition of $(\widehat{\beta}, \widehat{\gamma})$ in (1) and feasibility of $(\beta^\star, \gamma^\star)$, we have

$$\begin{aligned} &\mathcal{Q}_{S,T}(\widehat{\beta}, \widehat{\gamma}) \;\le\; \mathcal{Q}_{S,T}(\beta^\star, \gamma^\star) \\ \Rightarrow \quad &\frac{1}{ST} \sum_{s,t} \{\ell_{s,t}(\widehat{\beta}, \widehat{\gamma}) - \ell_{s,t}(\beta^\star, \gamma^\star)\} + \lambda_\beta\big(\|\widehat{\beta}\|_1 - \|\beta^\star\|_1\big) + \lambda_\gamma\big(\|\widehat{\gamma}\|_1 - \|\gamma^\star\|_1\big) \;\le\; 0. \end{aligned}$$

Define $\Delta_\beta = \widehat{\beta} - \beta^\star$ and $\Delta_\gamma = \widehat{\gamma} - \gamma^\star$, and write the empirical risk difference as

$$\underbrace{\{R(\widehat{\beta}, \widehat{\gamma}) - R(\beta^\star, \gamma^\star)\}}_{\text{population}} + \underbrace{\Big[\tfrac{1}{ST} \sum_{s,t} \big(\ell_{s,t}(\widehat{\beta}, \widehat{\gamma}) - \ell_{s,t}(\beta^\star, \gamma^\star)\big) - \big(R(\widehat{\beta}, \widehat{\gamma}) - R(\beta^\star, \gamma^\star)\big)\Big]}_{\text{empirical process}} \;\le\; -\lambda_\beta\big(\|\widehat{\beta}\|_1 - \|\beta^\star\|_1\big) - \lambda_\gamma\big(\|\widehat{\gamma}\|_1 - \|\gamma^\star\|_1\big).$$

Step 2: Controlling the empirical process term. By Assumptions S1 and S3, which provide smoothness and score concentration under weak dependence, the empirical process term

satisfies, on an event of probability at least $1 - C\exp(-c\log p)$,

$$\Big|\tfrac{1}{ST}\sum_{s,t}\big(\ell_{s,t}(\widehat{\beta},\widehat{\gamma}) - \ell_{s,t}(\beta^\star,\gamma^\star)\big) - \big(R(\widehat{\beta},\widehat{\gamma}) - R(\beta^\star,\gamma^\star)\big)\Big| \;\le\; \tfrac{\lambda_\beta}{2}\|\Delta_\beta\|_1 + \tfrac{\lambda_\gamma}{2}\|\Delta_\gamma\|_1,$$

for $\lambda_\beta, \lambda_\gamma \asymp \sqrt{(\log p)/(ST)}$ up to constants depending on c_0. Substituting this bound into the basic inequality yields

$$\begin{aligned} R(\widehat{\beta},\widehat{\gamma}) - R(\beta^\star,\gamma^\star) &\le \tfrac{\lambda_\beta}{2}\|\Delta_\beta\|_1 + \tfrac{\lambda_\gamma}{2}\|\Delta_\gamma\|_1 - \lambda_\beta\big(\|\widehat{\beta}\|_1 - \|\beta^\star\|_1\big) - \lambda_\gamma\big(\|\widehat{\gamma}\|_1 - \|\gamma^\star\|_1\big) \\ &\le \tfrac{3\lambda_\beta}{2}\|\Delta_{\beta,S_\beta}\|_1 + \tfrac{3\lambda_\gamma}{2}\|\Delta_{\gamma,S_\gamma}\|_1, \end{aligned}$$

where in the last inequality we used the usual cone constraints $\|\Delta_{\beta,S_\beta^c}\|_1 \le 3\|\Delta_{\beta,S_\beta}\|_1$ and similarly for γ, which follow from standard ℓ_1-penalized estimation arguments.

Step 3: Restricted strong convexity and oracle rate. By Assumption S2 (RSC), we have

$$R(\widehat{\beta},\widehat{\gamma}) - R(\beta^\star,\gamma^\star) \;\ge\; \kappa\big(\|\Delta_\beta\|_2^2 + \|\Delta_\gamma\|_2^2\big) - \tau\frac{\log p}{ST}.$$

Combining this with the upper bound from Step 2 and using the inequalities $\|\Delta_{\beta,S_\beta}\|_1 \le \sqrt{s_\beta}\|\Delta_\beta\|_2$ and $\|\Delta_{\gamma,S_\gamma}\|_1 \le \sqrt{s_\gamma}\|\Delta_\gamma\|_2$, we obtain

$$\kappa\big(\|\Delta_\beta\|_2^2 + \|\Delta_\gamma\|_2^2\big) \;\lesssim\; \lambda_\beta\sqrt{s_\beta}\|\Delta_\beta\|_2 + \lambda_\gamma\sqrt{s_\gamma}\|\Delta_\gamma\|_2 + \tau\frac{\log p}{ST}.$$

A standard quadratic inequality then yields

$$\|\Delta_\beta\|_2^2 + \|\Delta_\gamma\|_2^2 \;\lesssim\; (s_\beta + s_\gamma)\,\lambda^2 \;\asymp\; (s_\beta + s_\gamma)\,\frac{\log p}{ST},$$

where λ^2 stands for the common order of λ_β^2 and λ_γ^2. Substituting back into the RSC bound on the excess risk gives

$$R(\widehat{\beta},\widehat{\gamma}) - R(\beta^\star,\gamma^\star) \;\lesssim\; (s_\beta + s_\gamma)\,\frac{\log p}{ST},$$

which is exactly (2). This completes the proof. □

S2 Algorithmic Details

The main manuscript introduces the penalized panel ARX–GARCHX model and the exceedance-based risk score. This section gives the full estimation algorithm and the implementation details used for the simulation and WESAD analyses.

The estimator alternates between a mean block and a variance block via block coordinate descent. The mean block updates the subject-specific intercepts α_s in closed form and then the shared mean parameters (θ, β) via a weighted penalized regression step. The variance block is decoupled in two stages: a constrained nonlinear update for the GARCH parameters (ω, a, b) with γ fixed (B1), followed by a proximal-gradient update for the variance covariate effects γ with (ω, a, b) fixed (B2). A forward recursion then refreshes the conditional variance $\sigma^2_{s,t}$ for the next outer iteration. The full procedure is summarized in Algorithm 1.

Subject-specific intercepts. Given current conditional variance estimates, the subject-specific intercepts in the conditional mean equation are updated by weighted least squares:

$$\alpha_s \leftarrow \frac{\sum_t w_{s,t} \left(y_{s,t} - \sum_{i=1}^{P} \theta_i y_{s,t-i} - \sum_{j=0}^{Q} x_{s,t-j}^{\top} \beta_j \right)}{\sum_t w_{s,t}}, \qquad w_{s,t} = \sigma_{s,t}^{-2}. \tag{3}$$

A centering constraint $\sum_s \alpha_s = 0$ may be imposed for identifiability.

Convexity and positivity safeguards. Each block is convex once the others are held fixed. The mean block updates the subject-specific intercepts α_s in closed form and then the shared mean parameters (θ, β) via a weighted penalized regression step. The variance update is convex in the non-negative GARCH parameters (ω, a, b) for fixed γ (subblock B1) and convex in γ for fixed (ω, a, b) (subblock B2), since $u \mapsto \log u + c/u$ is convex on $(0, \infty)$. Positivity of the conditional variance is enforced by constraining $\omega_s > 0$, $a_r \geq 0$, and $b_\ell \geq 0$, and by projecting the variance recursion onto a small positive lower bound inside the log and ratio terms whenever the unconstrained linear predictor would otherwise approach zero.

Implementation choices used in this paper. Covariates were standardized within subject before model fitting. The primary analyses used lag orders $(P, Q, L, R, M) = (1, 0, 1, 1, 0)$, reflecting the short-memory structure of typical wearable sensor data; sensitivity checks with larger orders did not materially change the results. The penalties (λ, ρ) for the mean and variance blocks were selected by blockwise time-series cross-validation, partitioning each subject's

Algorithm 1 Penalized Panel ARX–GARCHX with Decoupled Variance Updates

Input: Panel $\{(y_{s,t}, x_{s,t})\}$; orders P, Q (mean), L, R, M (variance); penalties $P_\lambda(\beta), P_\rho(\gamma)$.
Init: $\Theta^{(0)} = (\alpha^{(0)}, \theta^{(0)}, \beta^{(0)})$, $\Phi^{(0)} = (\omega^{(0)}, a^{(0)}, b^{(0)}, \gamma^{(0)})$; compute $\sigma^{2,(0)}_{s,t}$.
for $k = 0, 1, \dots$ until convergence **do**
  (A) Mean block (weighted penalized regression).
  $w_{s,t} \leftarrow 1/\sigma^{2,(k)}_{s,t}$.
  $\alpha_s^{(k+1)} \leftarrow \dfrac{\sum_t w_{s,t}\big(y_{s,t} - \sum_i \theta_i^{(k)} y_{s,t-i} - \sum_j \beta_j^{(k)} x_{s,t-j}\big)}{\sum_t w_{s,t}}$.
  $\{\theta, \beta\}^{(k+1)} \in \arg\min_{\theta,\beta} \sum_{s,t} w_{s,t}\big(y_{s,t} - \alpha_s^{(k+1)} - \sum_i \theta_i y_{s,t-i} - \sum_j \beta_j x_{s,t-j}\big)^2 + P_\lambda(\beta)$.
  $\hat{e}^{(k+1)}_{s,t} \leftarrow y_{s,t} - \mu_{s,t}(\alpha^{(k+1)}, \theta^{(k+1)}, \beta^{(k+1)})$.
  Build strict mask
  $I = \{(s,t) : \text{all required lags of } y, e, \sigma^2, x \text{ exist}\}$.
  (B1) Variance sub-block: (ω, a, b) **given** $\gamma^{(k)}$**.**
  $\delta_{s,t}(\omega, a, b) := \omega_s + \sum_{r=1}^{R} a_r\, \hat{e}^{2,(k+1)}_{s,t-r} + \sum_{\ell=1}^{L} b_\ell\, \sigma^{2,(k)}_{s,t-\ell}$.
  $(\omega^\star, a^\star, b^\star) \in \arg\min_{\omega,\, a, b \geq 0,\, \sum a + \sum b \leq \phi_{\max}} \sum_{(s,t)\in I} \Big\{ \log u_{s,t} + \hat{e}^{2,(k+1)}_{s,t}/u_{s,t} \Big\}$,
  where $u_{s,t} = \delta_{s,t}(\omega, a, b) + x_{s,t}^\top \gamma^{(k)}$.
  (B2) Variance sub-block: γ **given** $\delta^\star$**.**
  With $\delta^\star_{s,t} = \delta_{s,t}(\omega^\star, a^\star, b^\star)$ fixed, $\gamma^{(k+1)} \in \arg\min_{\gamma} \sum_{(s,t)\in I} \Big\{ \log(\delta^\star_{s,t} + x_{s,t}^\top \gamma) + \hat{e}^{2,(k+1)}_{s,t}/(\delta^\star_{s,t} + x_{s,t}^\top \gamma) \Big\} + P_\rho(\gamma)$.
  (C) Forward recursion: $\sigma^{2,(k+1)}_{s,t} \leftarrow \delta^\star_{s,t} + x_{s,t}^\top \gamma^{(k+1)}$.
end for
Output: $\widehat{\alpha}_s, \widehat{\theta}, \widehat{\beta}, \widehat{\omega}_s, \widehat{a}, \widehat{b}, \widehat{\gamma}$.

windows into contiguous folds to preserve temporal ordering and aggregating validation losses across subjects. Convergence was declared when the relative change in the full objective fell below a prespecified tolerance or after the maximum number of outer iterations (20 in the WESAD analysis), at which point fewer than 15 outer iterations were typically sufficient.

S3 Supplementary Simulation Results

Table S1 reports the replication-level Bias and RMSE of the estimated exceedance risk for all six competing models. Columns A–D are the original variants; **Model E** (ARX_p–GARCHX; penalized mean, unpenalized X-dependent variance) and **Model F** (AR–GARCH; no covariates in the mean or variance) were added in response to review. We compute E and F under the identical generator, evaluator, tuning, and seed scheme as the reported A–D run (100 replications), so that Columns A–D are unchanged and the two new columns are directly comparable.

S4 Supplementary Computational Results

Table S2 reports the runtime profile for the WESAD analysis. These details are provided in the supplement so that the main manuscript remains focused on the methodology and translational demonstration.

Table S2: Computational characteristics of the temporal risk-score model in the WESAD analysis.

**Component**	**Metric**	**Value**
Dataset scale	(S, T, d)	$(15, 120, 77)$
Mean update	Time per iteration	≈ 0.01 s
Variance update	Time per iteration	≈ 0.12 s
Total training	End-to-end runtime	≈ 26.6 s for 20 iterations
Inference	Latency for one new window	< 1 ms
Asymptotic complexity	Mean and variance recursions	$O(STd)$ and $O(ST(L+R+M))$
Memory footprint	Space complexity	$O(ST+d)$

Timings were obtained on a MacBook Air with an M2 processor and eight GB RAM. These timings document practical feasibility for the WESAD benchmark rather than optimized software performance.

Table S1: Bias and RMSE of estimated exceedance risk (means and standard errors in parentheses) across simulation Scenarios 1–4 with 100 replications, for the six competing models.

(a) Bias

T=120

	**Model**					
**Scenario**	A<br>ARX–GARCH	B<br>ARXp–GARCH	C<br>ARX–GARCHX	D<br>ARXp–GARCHXp	E<br>ARXp–GARCHX	F<br>AR–GARCH
1	-0.001 (0.001)	-0.002 (0.001)	0.004 (0.001)	0.005 (0.002)	-0.003 (0.001)	-0.001 (0.001)
2	-0.002 (0.000)	-0.002 (0.001)	-0.001 (0.000)	-0.001 (0.001)	-0.002 (0.001)	-0.010 (0.002)
3	-0.000 (0.002)	-0.000 (0.002)	0.005 (0.002)	0.004 (0.001)	-0.002 (0.001)	-0.002 (0.001)
4	-0.005 (0.000)	-0.005 (0.000)	0.001 (0.001)	-0.000 (0.001)	-0.004 (0.000)	-0.015 (0.002)

T=240

	**Model**					
**Scenario**	A<br>ARX–GARCH	B<br>ARXp–GARCH	C<br>ARX–GARCHX	D<br>ARXp–GARCHXp	E<br>ARXp–GARCHX	F<br>AR–GARCH
1	-0.001 (0.001)	-0.001 (0.001)	0.014 (0.001)	0.014 (0.001)	-0.002 (0.000)	-0.003 (0.002)
2	-0.000 (0.000)	-0.001 (0.000)	0.001 (0.000)	0.000 (0.001)	-0.002 (0.000)	-0.006 (0.001)
3	-0.002 (0.000)	-0.003 (0.001)	0.012 (0.001)	0.012 (0.001)	-0.004 (0.000)	-0.002 (0.002)
4	-0.005 (0.000)	-0.005 (0.000)	0.001 (0.000)	0.000 (0.001)	-0.005 (0.000)	-0.015 (0.001)

(b) RMSE

T=120

	**Model**					
**Scenario**	A<br>ARX–GARCH	B<br>ARXp–GARCH	C<br>ARX–GARCHX	D<br>ARXp–GARCHXp	E<br>ARXp–GARCHX	F<br>AR–GARCH
1	0.085 (0.001)	0.084 (0.001)	**0.080 (0.001)**	0.082 (0.003)	0.082 (0.002)	0.161 (0.002)
2	0.072 (0.001)	0.073 (0.002)	0.064 (0.001)	**0.063 (0.002)**	0.068 (0.002)	0.107 (0.003)
3	0.096 (0.003)	0.093 (0.003)	0.087 (0.003)	**0.084 (0.002)**	0.090 (0.003)	0.133 (0.001)
4	0.093 (0.000)	0.091 (0.000)	0.094 (0.001)	0.093 (0.002)	**0.089 (0.001)**	0.127 (0.002)

T=240

	**Model**					
**Scenario**	A<br>ARX–GARCH	B<br>ARXp–GARCH	C<br>ARX–GARCHX	D<br>ARXp–GARCHXp	E<br>ARXp–GARCHX	F<br>AR–GARCH
1	0.079 (0.002)	0.077 (0.002)	0.076 (0.001)	0.078 (0.003)	**0.070 (0.002)**	0.163 (0.003)
2	0.063 (0.001)	0.062 (0.001)	0.056 (0.001)	0.060 (0.002)	**0.054 (0.001)**	0.097 (0.002)
3	0.083 (0.001)	0.082 (0.001)	0.081 (0.001)	0.080 (0.001)	**0.076 (0.001)**	0.131 (0.002)
4	0.084 (0.000)	0.083 (0.000)	0.084 (0.000)	0.083 (0.002)	**0.075 (0.001)**	0.128 (0.001)

S5 WESAD Endpoint and Per-Participant Composition

The reference-based evaluation in Section 4.3 of the main text uses a binary window-level endpoint. The positive class is the set of windows annotated *stress* (WESAD label 2); the negative class pools all remaining retained windows, namely baseline (label 1), amusement (label 3), and transition/other (label 0). Transition and amusement windows are thus retained and treated as non-stress rather than discarded, so that the denominator reflects the full recording. After preprocessing (Section S8), 1,427 windows from 15 participants remained, of which 182 are stress and 1,245 non-stress. Table S3 gives the composition for each participant.

Table S3: Per-participant window and class counts in the WESAD analysis. "Non-stress" pools baseline, amusement, and transition/other windows.

Subject	Baseline	Stress	Amusement	Transition/other	Total	Stress (pos.)	Non-stress (neg.)
2	20	12	7	61	100	12	88
3	20	12	7	68	107	12	95
4	21	11	7	67	106	11	95
5	21	11	7	64	103	11	92
6	21	12	7	76	116	12	104
7	21	12	8	45	86	12	74
8	21	13	7	49	90	13	77
9	20	11	7	48	86	11	75
10	21	13	7	49	90	13	77
11	21	13	7	45	86	13	73
13	21	12	8	50	91	12	79
14	20	12	8	51	91	12	79
15	21	13	8	44	86	13	73
16	21	12	7	52	92	12	80
17	21	13	7	56	97	13	84
**Total**	311	182	109	825	1427	182	1245

Because the 1,427 windows are clustered within only 15 participants, a pooled DeLong test that treats windows as independent overstates precision. We therefore additionally quantify uncertainty with a *participant-clustered* bootstrap that resamples whole participants with replacement (3,000 resamples) and recomputes the pooled AUC on each resample. Re-scoring the saved model fit reproduces the AUCs reported in the main text (risk score 0.931, raw `HR_mean` 0.868) to within 0.005 (0.936 and 0.870). The participant-clustered 95% confidence intervals are [0.887, 0.972] for the risk score and [0.821, 0.918] for `HR_mean`. For the paired difference the interval is [0.031, 0.094], which excludes zero: the improvement of the variance-aware risk score over the raw heart rate is robust to within-participant clustering.

S6 Sensitivity to the Threshold Choice (Information-Leak Check)

The threshold $c_s(0.70)$ used in the main analysis is the 70th percentile of each participant's `HR_mean` over the *entire* recording. Although this uses no stress labels, it does include the high-

heart-rate windows that later form the positive class, which could lend a modest optimism to the reported discrimination. To assess this, we recomputed the exceedance score using thresholds set without such information—from an early per-participant burn-in window (the first 20% or 30% of each participant's windows) and, for reference, from that participant's baseline-annotated windows only—while holding the fitted conditional mean and variance fixed. Results are summarized in Table S4 and Figure S1.

Table S4: Discrimination of the annotated stress state (AUC) as a function of how the subject-specific threshold $c_s(0.70)$ is set. The conditional mean and variance are unchanged; only the threshold differs.

Rule for setting $c_s(0.70)$	AUC (stress vs. non-stress)
Full timeframe (main analysis)	0.936
Burn-in: first 20% of windows	0.876
Burn-in: first 30% of windows	0.886
Baseline-annotated windows only	0.870
Raw `HR_mean` baseline (reference)	0.870

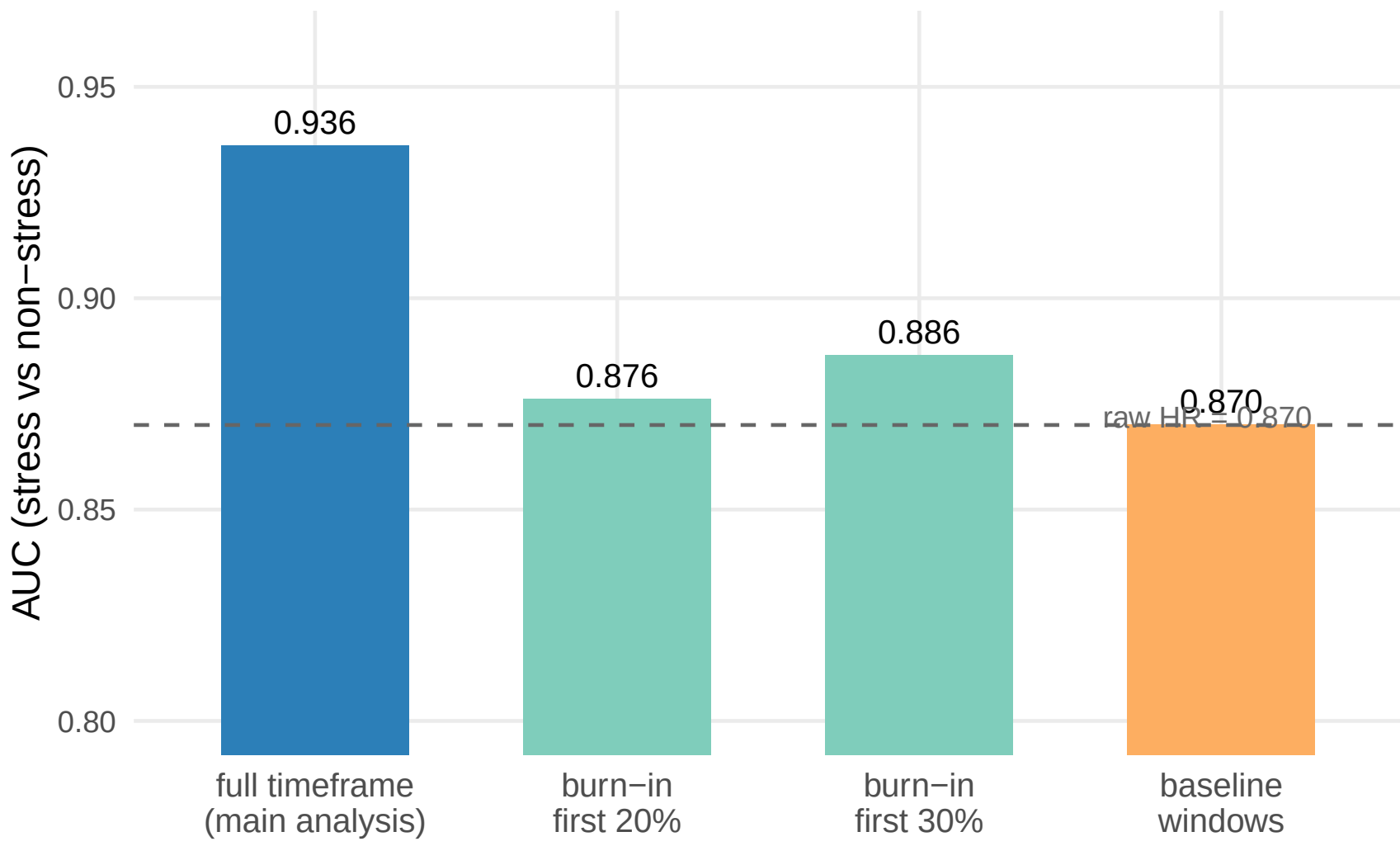

Figure S1: Risk-score AUC by the rule used to set the subject-specific threshold $c_s(0.70)$. A leak-free burn-in threshold reduces the AUC from 0.936 to approximately 0.88, which remains at or above the raw `HR_mean` baseline (dashed line).

Using a leak-free burn-in threshold reduces the AUC from 0.936 to approximately 0.88, still at or above the raw `HR_mean` baseline of 0.870. The temporal signal captured by the variance-aware score is therefore genuine, but the exact headline value depends on how the threshold is set; for prospective use the threshold should be fixed from an initial calibration period, as discussed in Section 5 of the main text.

S7 WESAD Variance-Covariate Ablation (Model B vs. Model D)

To isolate the contribution of covariate-driven volatility to the real-data results, we compare the full model (Model D, ARX_p–GARCHX_p: covariates in both the mean and the variance) with an otherwise identical model that removes covariates from the variance equation (Model B, ARX_p–GARCH). Both share the same penalized mean ($\lambda_\beta = 0.01$); they differ only in whether $x_{s,t}$ enters the conditional variance. We report discrimination of the annotated stress state (AUC) and, for the *actual* exceedance event $\{y_{s,t} > c_s(0.70)\}$ that the score is designed to predict, its discrimination (AUC) and calibration (Brier score). To keep the comparison controlled, both models are refit from scratch under identical settings in a single run; Model D here is the paper's main WESAD model, and its values differ from the headline AUC of 0.931 only through independent refitting.

Table S5: WESAD ablation. Discrimination of the annotated stress state and discrimination/calibration of the actual exceedance event $\{y > c_s(0.70)\}$ for the full model (D) and the no-variance-covariate model (B).

Model	AUC (stress)	AUC (event $y > c_s$)	Brier (event)
D: ARX_p–GARCHX_p (X in variance)	0.921	0.927	0.085
B: ARX_p–GARCH (no X in variance)	0.934	0.929	0.084

Two conclusions follow. First, the variance-aware exceedance score—the central contribution—discriminates the annotated stress state far better than the raw heart rate (both models near 0.93 versus 0.868 for `HR_mean`) and is well calibrated for the exceedance event it is designed to predict (Brier $\approx$ 0.085, event rate 0.301). Second, whether covariates enter the *variance* equation makes little practical difference on WESAD: Models B and D differ by at most 0.013 in AUC and 0.001 in Brier, which is an order of magnitude smaller than the participant-level sampling uncertainty (the participant-clustered 95% interval for a single AUC spans roughly ± 0.045; Section S5), so the two are statistically indistinguishable here. This is consistent with the simulation study, where covariate-driven variance helps specifically when the volatility is covariate-structured (Scenarios 2–4): WESAD heart-rate volatility appears to be captured adequately by the GARCH dynamics alone, so the additional variance covariates neither help nor hurt materially. The exceedance score is therefore robust to this modeling choice, and Model D is retained as the general specification that also accommodates the covariate-driven-volatility

regimes demonstrated in simulation.

S8 Reproducibility Details for the WESAD Analysis

Features. Windowed features were computed per 60-s window (60-s hop) from the chest-worn RespiBAN streams. Moment- and correlation-based construction produced a larger candidate set, which was reduced to the 77 used for modeling by removing one member of each pair with absolute Pearson correlation above 0.90 (`caret::findCorrelation`). The complete list of the 77 retained features, grouped by sensing block, is given in Table S6.

Table S6: Complete list of the 77 chest-worn features used in the WESAD model, grouped by sensing block (counts in parentheses).

**Block**	**Features**
**ECG / HRV** (32)	HRV_ApEn, HRV_C1a, HRV_CD, HRV_CSI_Modified, HRV_CVI, HRV_Ca, HRV_DFA_alpha1, HRV_FuzzyEn, HRV_HF, HRV_HFD, HRV_HFn, HRV_HTI, HRV_KFD, HRV_LF, HRV_LFHF, HRV_LZC, HRV_LnHF, HRV_MCVNN, HRV_MinNN, HRV_PAS, HRV_PI, HRV_PSS, HRV_S, HRV_SD1SD2, HRV_SD2d, HRV_SDRMSSD, HRV_SI, HRV_SampEn, HRV_ShanEn, HRV_TINN, HRV_pNN20, HRV_pNN50
**Respiration & BRV** (9)	BRV_CV, BRV_RMSSD, Duty_cycle, ExpDur_mean, InspDur_mean, RSP_amp_mean, RSP_amp_std, RSP_rate_mean, RSP_rate_std
**EDA** (9)	EDA_kurt, EDA_mean, EDA_skew, SCL_CV, SCL_slope_cpm, SCL_std, SCR_amp_mean, SCR_area, SCR_count_pm
**EMG** (4)	EMG_MDF, EMG_Power, EMG_SpecEnt, EMG_ZCR
**Accelerometry** (9)	ACC_kurt, ACC_skew, ACC_std, ACCjerk_RMS, ACCmag_P90, ACCmag_RMS, ACCx_med, ACCy_med, ACCz_med
**Skin temperature** (4)	TEMP_detrended_std, TEMP_mean, TEMP_skew, TEMP_slope_cpm
**Cross-channel correlations** (10)	corr_ACC4_EMG4_4hz, corr_ACC4_TEMP4_4hz, corr_RSP4_ACC4_4hz, corr_RSP4_EMG4_4hz, corr_RSP4_SCL4_4hz, corr_RSP4_TEMP4_4hz, corr_SCL4_ACC4_4hz, corr_SCL4_EMG4_4hz, corr_SCL4_TEMP4_4hz, corr_TEMP4_EMG4_4hz

Windows, missingness, and the panel. A window was retained only if at least 80% of its samples were finite in every required channel; windows with any non-finite feature after construction were dropped and no imputation was used. The retained window count for each

participant is given in Table S3 (range 86–116; participants S1 and S12 are excluded following the WESAD convention). Because participants contribute different numbers of windows, the analyzed panel is *unbalanced*. The balanced-panel notation of the main text is expositional only: the estimator builds all y-, residual-, variance-, and covariate-lags *within* each participant (Algorithm 1), so unequal series lengths are handled directly, without truncation or padding, and only the shared parameters $(\theta, \beta, a, b, \gamma)$ are pooled across participants.

Tuning grid and temporal folds. Penalties were selected to preserve temporal ordering: each participant's windows were split into contiguous (non-shuffled) time-series folds, and validation losses were aggregated across participants (blockwise time-series cross-validation). In the simulations, λ_β was searched on a multiplicative grid around 5×10^{-3} and λ_γ on a logarithmically spaced grid refined around its minimizer. For the WESAD analysis the selected values were $\lambda_\beta = 1 \times 10^{-2}$ (mean) and $\lambda_\gamma = 1 \times 10^{-4}$ (variance), with fixed lag orders $(P, Q, L, R, M) = (1, 0, 1, 1, 0)$ and 20 outer iterations.

Software and code. Preprocessing and feature extraction used Python with NeuroKit2 (ECG/PPG/EDA/respiration features). Model fitting and evaluation used R with `glmnet` (penalized updates), `pROC` (ROC/AUC and DeLong test), and `randomForest` and `gbm` (supervised comparators); collinearity pruning used `caret`. Exact package versions and the code release (commit hash) used for the reported results are: R 4.5.3, with `glmnet` 5.0, `pROC` 1.19.0.1, `randomForest` 4.7.1.2, `gbm` 2.2.3, and `caret` 7.0.1; feature extraction used Python with NeuroKit2. The exact analysis code is released at the repositories given in the main-text Code Availability statement. The `varGuidTS` package and the replication scripts are available at the repositories listed in the main-text Code Availability statement.
